# Supplementary material for: Bacteriostatic Effects of Yujin Powder and Its Components on Clinical Isolation of Multidrug-Resistant Avian Pathogenic Escherichia coli
Source: Vet Sci. 2023 May 4;10(5):328. doi: 10.3390/vetsci10050328 (PMC10223929; doi:10.3390/vetsci10050328)
Supplement: Supplementary file 1 [file vetsci-10-00328-s001.zip › vetsci-2303253-supplementary.pdf]

Bacteriostatic Effects of Yujin Powder and its Components on Clinical Isolation of Multidrug Resistant Avian Pathogenic Escherichia coli

Supplementary materials

Supplementary method 1: Numbers of the isolate after the treatment of drugs

The bacteria were added to the solution diluted with MH broth, so that the final concentration of bacteria was 1×10^5 CFU/mL and the final concentration of each drug is 1/2 MIC. The bacterial amounts were counted by plate colony counting after shaking culture at 37 °C for 24 h. The bacterial solutions were diluted 10 times, and dripped to the surface of nutrient agar plate. The plates were incubated at 37 °Cfor 24 h. Count the number of colonies between 10 and 70 and calculate the bacterial amounts. Each dilution was repeated for 3 times.

Supplementary methods 2: Determination of median lethal dose of isolated MDREC

ICR mice weighed 18~20 g, half male and half female, were fed adaptively for 7 days. They were randomly divided into 6 groups with 10 mice in each group. The mice were intraperitoneally injected with different concentrations of isolated bacteria at 0.2 mL/20 g. The control group was intraperitoneally injected with equal volume of sterilized normal saline. The clinical symptoms and death of mice were observed and recorded for 7 d continuously.

Supplementary Table S1 The numbers of colony-forming units of MDREC after 24 hours

Groups	Concentrations（mg/mL）	Total number of colony-forming units (CFU/mL)
BC	——	1.60×10^{9}
YJP	125.0（1/2MIC）	1.60×10^{9}
Rb	125.0(1/2MIC)	1.50×10^{7}
SR	31.25(1/2MIC)	6.50×10^{8}
Bac	1.0(1/2MIC)	1.95×10^{9}

BC: Blank Control; YJP: Yujin Powder; Rb: Rhubarb; SR: Scutellariae Radix; Bac: Baicalin.

Supplementary Table S2 The death situation of mice caused by different amounts of infected bacteria

Group	Number of bacteria (CFU/mL)	Sample Number	Death number	Death rate (%)
A	7.25×10^8	10	10	100
B	5.80×10^8	10	9	90
C	4.30×10^8	10	6	60
D	3.75×10^8	10	6	60
E	2.95×10^8	10	0	0
BC	0	10	0	0

A: The mice inoculated with 7.25×10^8 CFU/mL bacteria. B: The mice inoculated with 5.80×10^8 CFU/mL bacteria. C: The mice inoculated with 4.30×10^8 CFU/mL bacteria D: The mice inoculated with 3.75×10^8 CFU/mL bacteria. E: The mice inoculated with 2.95×10^8CFU/mL bacteria. BC: Black control, the mice inoculated with equivalent physiological saline.
